# Light-Induced Nonthermal Phase Transition to the Topological Crystalline Insulator State in SnSe

**DOI:** 10.1021/acs.jpclett.3c02450

**Published:** 2023-10-11

**Authors:** Stefano Mocatti, Giovanni Marini, Matteo Calandra

**Affiliations:** Department of Physics, University of Trento, Via Sommarive 14, 38123 Povo, Italy

## Abstract

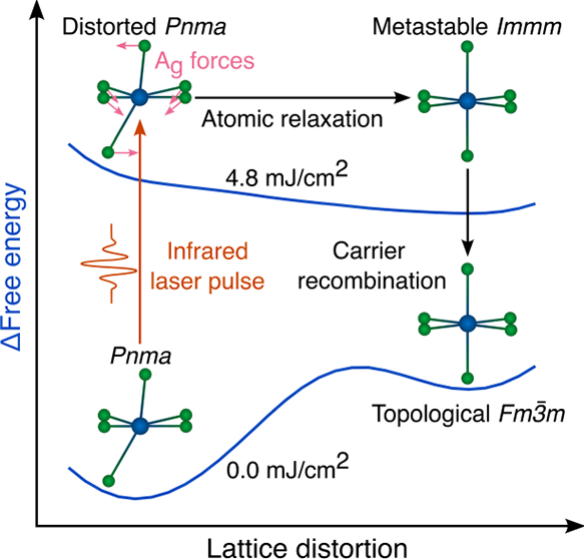

Femtosecond pulses have been used to reveal hidden broken symmetry states and induce transitions to metastable states. However, these states are mostly transient and disappear after laser removal. Photoinduced phase transitions toward crystalline metastable states with a change of topological order are rare and difficult to predict and realize experimentally. Here, by using constrained density functional perturbation theory and accounting for light-induced quantum anharmonicity, we show that ultrafast lasers can permanently transform the topologically trivial orthorhombic structure of SnSe into the topological crystalline insulating rocksalt phase via a first-order nonthermal phase transition. We describe the reaction path and evaluate the critical fluence and possible decay channels after photoexcitation. Our simulations of the photoexcited structural and vibrational properties are in excellent agreement with recent pump–probe data in the intermediate fluence regime below the transition with an error on the curvature of the quantum free energy of the photoexcited state that is smaller than 2%.

The development of ultrafast laser light with femtosecond (fs) pulses has led to the possibility of inducing a substantial electron–hole population unbalance in semiconductors.^1^ After some tens of femtoseconds, this electron–hole plasma is well described by a two-chemical potential model, where both electrons and holes are characterized by a thermal distribution. Thus, the ions feel an out-of-equilibrium electronic population with a substantial occupation of conduction or antibonding states that can lead to structural phase transitions before electron–hole recombination takes place. In this scenario, ultrafast pulses can be used to overcome free-energy barriers and synthesize crystal structures that cannot be reached by conventional thermodynamical paths. This kind of structural transformation is labeled *nonthermal*, to distinguish it from the much slower ones involved in conventional (*thermal*) material synthesis. Experimental demonstrations of nonthermal phenomena induced by fs pulses are order–disorder phase transitions,^2^ charge density waves,^3^ nonthermal melting of solids,^4^ transient topological phase transitions^5^ and light-induced suppression of incipient ferroelectricity.^6^ In all of these cases, ultrafast light induces short-lived transient states. Much less common are light-induced *nonthermal* permanent structural modifications. In this work, we show that nonthermal processes can be used to permanently stabilize topological crystalline insulating (TCI) phases.^7−11^ In this work, we will focus our attention on tin selenide (SnSe), a IV–VI p-type narrow gap semiconductor that has become popular due to its attractive thermoelectric properties^12−15^ (*zT* = 2.6 at *T* = 923 K). At ambient conditions, tin selenide crystallizes in the orthorhombic *Pnma* structure, as sketched in Figure 1. At *T* ≈ 813 K^16^ or finite pressure,^17^ it undergoes a second-order phase transition to an orthorhombic *Cmcm* structure.^17−20^ In SnSe the topological nontrivial state occurs neither in the *Pnma* phase, nor in the *Cmcm* phase, but in a metastable rocksalt structure, which cannot be reached via a thermal transition, but it can only be synthesized in thin films via epitaxial growth techniques on a cubic substrate.^21^

**Figure 1:**
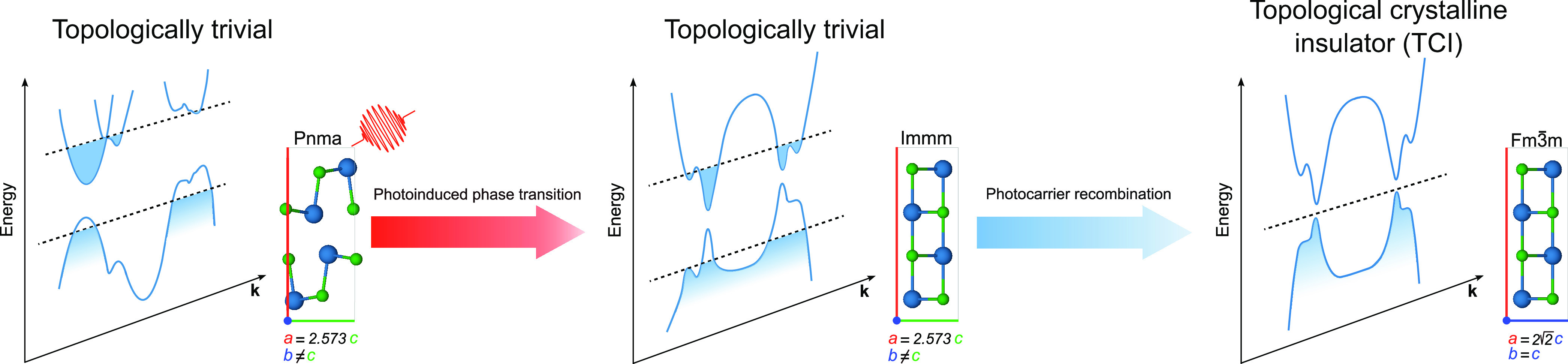
Pictorial representation of the nonthermal pathway connecting the topologically trivial *Pnma* structure with the TCI *Fm*3̅*m* structure. For each phase, sketched crystal structures and band structures are represented. Fs pulses induce a first-order transformation toward a transient phase with *Immm* symmetry. This phase spontaneously decays into the *Fm*3̅*m* structure after electron–hole recombination.

Here we design a different approach to obtain the topological crystalline phase of SnSe (see Figure 1), namely we consider the effect of ultrafast pulses on the topologically trivial *Pnma* phase, which is close to a band inversion.^22^ By laser pumping with a near-infrared pulse (1.55 eV) and monitoring the time evolution with time-resolved Raman and X-ray diffraction, it was recently shown that structural modifications occur in SnSe, signaled by *A*_*g*_ modes softening and fluence-dependent atomic displacements,^23^ interpreted as the precursor of a symmetrization toward a different orthorhombic structure with *Immm* symmetry. However, no transition to this new crystal phase was detected, and first-principles simulations were unable to reproduce the observed structural distortion.

In this Letter, we investigate the nonthermal structural transformations of the *Pnma* structure after irradiation with fs pulses by combining constrained density functional perturbation theory (c-DFPT)^24^ and stochastic self-consistent harmonic approximation (SSCHA),^25^ accounting for quantum anharmonicity in the presence of an electron–hole plasma for the first time. Further technical details are provided in the Supporting Information, which includes refs (26−43). We identify the nonthermal pathway (see Figure 1) and the critical fluence for the structural transition from the ground-state *Pnma* to the transient *Immm* phase. Our calculated structural distortions and softenings of the *A*_*g*_ modes along the reaction path are in excellent agreement with experimental data.^23^ Most importantly, we show that the transient *Immm* phase spontaneously decays into the TCI rocksalt *Fm*3̅*m* SnSe structure after electron–hole recombination and that the structural transformation is permanent by virtue of the free-energy barrier between the rocksalt and the *Pnma* phase.

In Figure 2a,b we display the optimized tin Wyckoff *x* and *z* coordinates as functions of the photocarrier concentration (PC), *n*_*e*_, expressed as the number of photoexcited electrons per unit cell (u.c.). Our results are compared with time-resolved diffraction data from ref (23) (see also Supporting Information, Section S3) measured in the first 5 ps after illumination. After photoexcitation, both the internal equilibrium positions and lattice parameters can change. However, the time scale for the two phenomena is generally different.^44^ To unambiguously disentangle cell deformation and internal displacements at a fixed cell, we perform structural optimization at a fixed cell, Figure 2a, and at a variable cell, Figure 2b, in the presence of an electron–hole plasma (the procedure regarding fluence/PC mapping is reported in the Supporting Information).

**Figure 2:**
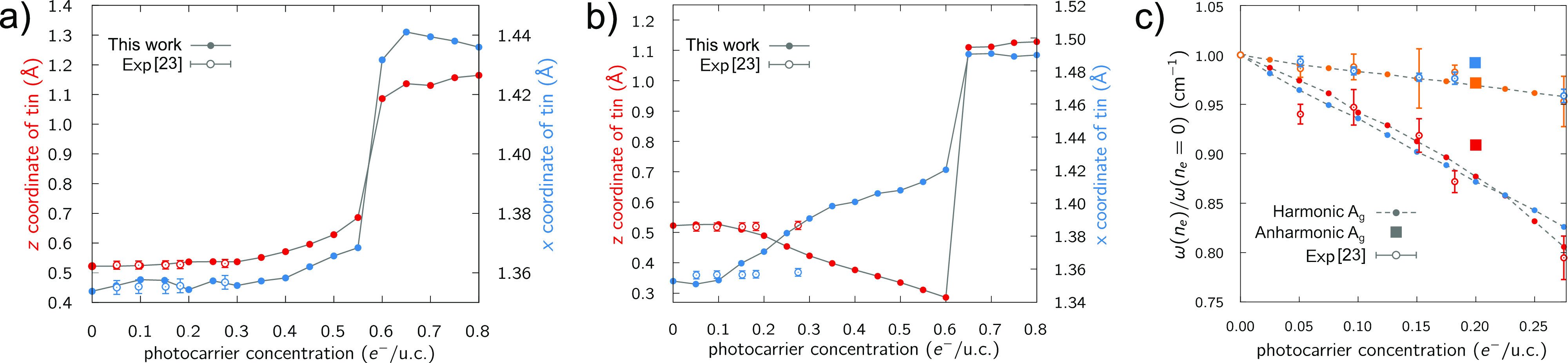
Tin Wyckoff positions *x* and *z* as functions of the photocarrier concentration for fixed (a) and variable (b) volume crystal structure optimization. The red and blue dots are labeled with the *z* and *x* coordinates of tin, respectively. (c) Normalized phonon frequencies at Γ for the three relevant *A*_*g*_ modes versus PC. The red, blue, and orange dots stand for the *A*_*g*,1_, *A*_*g*,2_, and *A*_*g*,4_ modes, respectively. The theoretical harmonic, anharmonic, and experimental values of ω_0_ are reported in the Supporting Information. The experimental data are from ref (23).

Our calculation at a fixed cell is in excellent agreement with time-resolved X-ray diffraction data in the first 5 ps, while that at a variable cell substantially deviates. This confirms that in the first picoseconds after irradiation the atoms are displaced at a fixed cell. Previous calculations from ref (23) (see Figure S5) obtained Sn displacements 1 order of magnitude larger than the experimental ones. Conversely, we report an excellent agreement between our c-DFT calculations and experimental data within our framework.^24^ The stark disagreement among the calculation of ref (23) and experiments arises because in ref (23) the electron and hole occupations are not self-consistently relaxed. This procedure does not lead to a correctly thermalized quasi-equilibrium Fermi–Dirac distribution. An explanation of the main differences between the approach of ref (23) and the c-DFT approach of refs (45 and 24) used in this work is reported in Section S3.1 in the Supporting Information.

The discontinuity in the *z*_Sn_ and *x*_Sn_ in Figure 2a,b at *n*_*e*_^*c*^ ≈ 0.6*e*^–^/ u.c. signals the occurrence of a first-order phase transition. The phase transition can be easily identified by noting that for *n*_*e*_ ≥ 0.6 *e*^–^/u.c. the Wyckoff positions of tin correspond to that of the *Immm* structure, where they are fixed by symmetry. Thus, we predict the phase transition from *Pnma* to *Immm* to occur at a value of *n*_*e*_ that is approximately a factor of 2 larger than the highest photocarrier concentration measured in ref (23).

Additional validation of our findings arises from the *A*_*g*_ harmonic and anharmonic phonon frequency calculation at a fixed cell. The results are shown in Figure 2c where they are compared with the measured frequencies of oscillation of the Bragg peaks in the first ps after pumping as a function of *n*_*e*_.^23^ We plot the value of the harmonic *A*_*g*_ phonon frequencies (full circles) at a given photocarrier concentration (i.e., ω(*n*_*e*_)) divided by the harmonic phonon frequency in the ground state (i.e., ω_0_ = ω(*n*_*e*_ = 0)). The softening of harmonic modes *A*_*g*,1_ and *A*_*g*,4_ induced by the photoexcitation is in excellent agreement with the experimental data. Concerning the *A*_*g*,2_ mode, c-DFPT overestimates the softening induced by the electron–hole plasma within the harmonic approximation. A possible reason for this discrepancy is the presence of strong anharmonic renormalization for the *A*_*g*,2_ mode. Thus, we calculated the anharmonic phonon frequencies in the absence of photocarriers and for *n*_*e*_ = 0.2 *e*^–^/u.c. at *T* = 0 K. Our results are depicted in Figure 2c, where the values of the normalized anharmonic phonon frequencies (ω(*n*_*e*_)/ω_0_) are represented as filled squares. The anharmonic corrections to the phonon frequencies of the *A*_*g*,1_ and *A*_*g*,4_ modes are mild and do not change the overall trend obtained at the harmonic level. On the contrary, the *A*_*g*,2_ mode is substantially affected by anharmonicity, resulting in an improved agreement with the experimental data. The maximum relative error in the predicted quantum phonon frequency softening is roughly 2%.

The *A*_*g*,2_ mode plays a crucial role in the *Pnma* → *Immm* phase transition. The observed strong anharmonic renormalization of the *A*_*g*,2_ mode indicates that the Born–Oppenheimer free-energy surface surrounding the minimum energy state corresponding to the *Pnma* phase becomes progressively more anharmonic along the path of the transition.

The crucial role of quantum anharmonicity becomes even more evident if the electronic structures together with the harmonic and quantum anharmonic dispersions of the *Pnma* and *Immm* phase are considered (in Figure 3). The insulating ground state (Figure 3a) displays a finite electronic gap (∼0.52 eV) and dynamically stable harmonic phonons (Figure 3c). The quantum anharmonic corrections on the phonon spectrum are essentially negligible. On the contrary, the light-induced *Immm* phase in the presence of an electron–hole plasma at *n*_*e*_ = 0.6 *e*^–^/u.c. has a metallic electronic structure with electron and hole Fermi surfaces located close to the high-symmetry Y and Z points (Figure 3b). These Fermi surfaces are nested (see the Supporting Information, Section S3) and trigger the emergence of a Peierls instability at the harmonic level signaled by imaginary phonons along the Γ–*X* direction at a wave-vector compatible with the nesting condition (Figure 3d). Structural minimization shows the emergence of a 2 × 1 × 1 one-dimensional chain-like charge density wave with an energy gain of ∼1.5 meV/atom with respect to the undistorted structure (see the Supporting Information). When quantum-anharmonic corrections are included within the SSCHA at *n*_*e*_ = 0.6 *e*^–^/u.c., we find that the instability is removed and a sharp one-dimensional Kohn-anomaly appears (Figure 3d). Thus, light-induced quantum anharmonicity stabilizes the *Immm* phase in the transient state at *n*_*e*_ = 0.6 *e*^–^/u.c.

**Figure 3:**
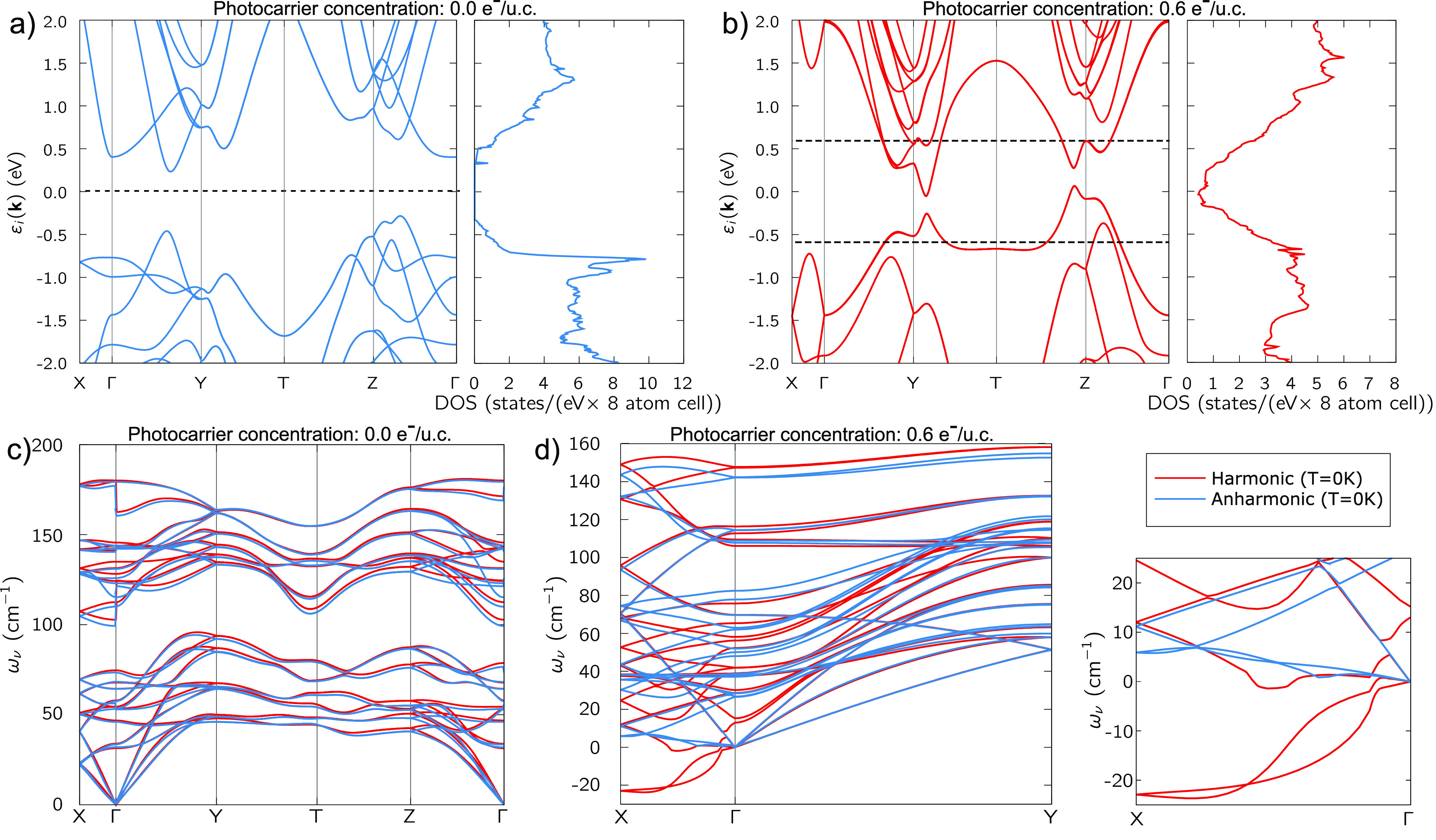
Ground-state electronic structure of the *Pnma* phase (a) and of the *Immm* transient phase at *n*_*e*_ = 0.6 *e*^–^/u.c. (b). The Fermi level in panel a and the holes and electron Fermi levels in panel b are depicted as dashed lines. Harmonic and anharmonic phonon spectra for the *Pnma* phase at *n*_*e*_ = 0.0 *e*^–^/u.c. (c) and for the transient *Immm* phase at *n*_*e*_ = 0.6 *e*^–^/u.c. (d). Both plots are at *T* = 0 K. The inset shows the removal of the dynamic instability by quantum anharmonic effects.

The critical PC of *n*_*e*_ = 0.6 *e*^–^/u.c., corresponding to ∼4.8 mJ/cm^2^, is achievable in ultrafast experiments; similar or larger values have already been achieved in narrow gap semiconductors without inducing significant damage to the sample.^46−48^

Having demonstrated the accuracy of our approach to describe the structural evolution after photoexcitation, we now try to understand more in detail the reaction path and the nature of this transition. In Figure 4, we display the energy along the paths relative to the (a) *Pnma* → *Immm* and (b) *Pnma* → *Cmcm* transitions, for a few values of *n*_*e*_. The path is parametrized by the reaction coordinate η, where η = 0 stands for the *Pnma* structure while η = 1 represents either the *Immm* or *Cmcm* structure.

**Figure 4:**
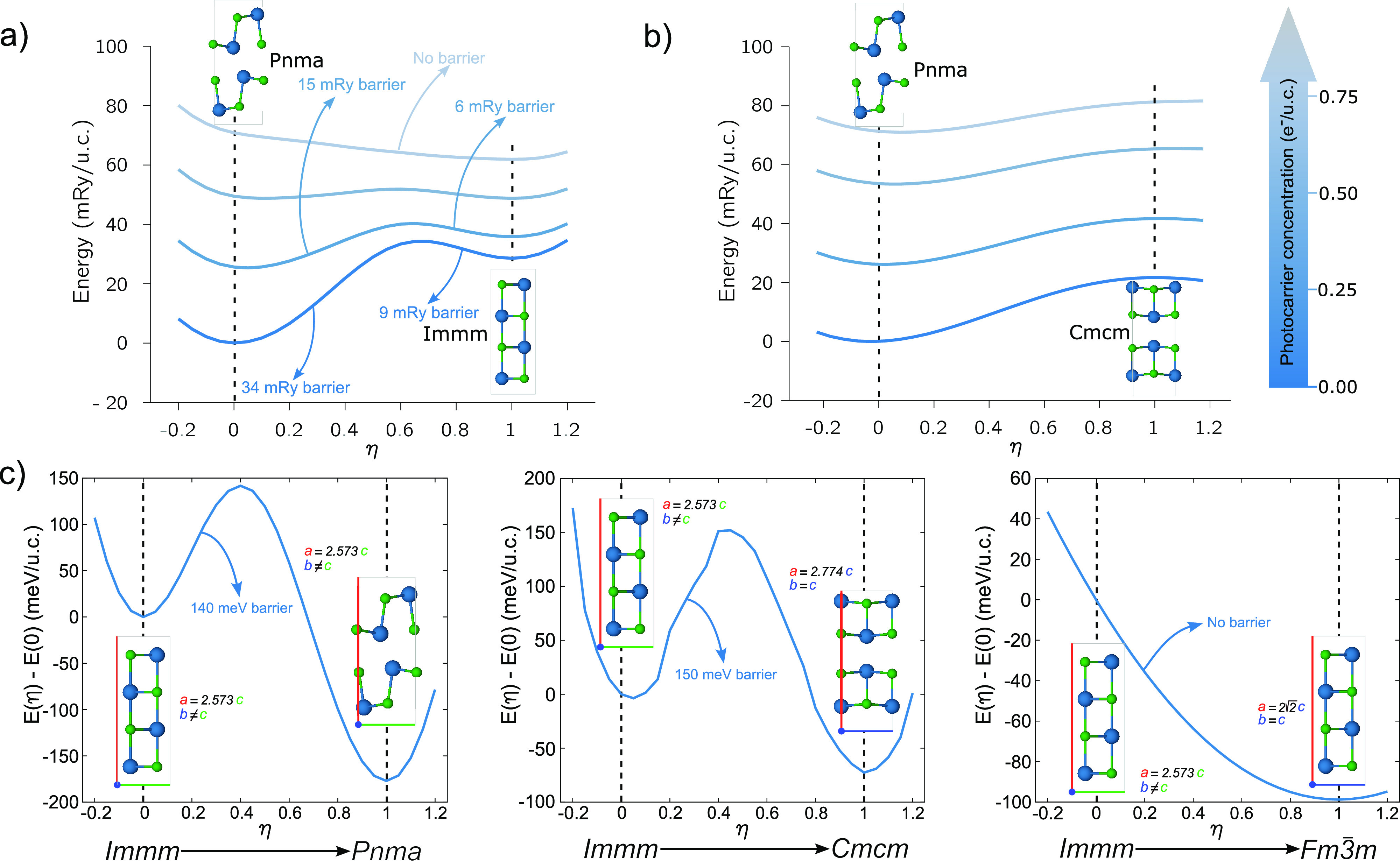
Total energy curves along the *Pnma* → *Immm* (a) and *Pnma* → *Cmcm* (b) reaction paths: η = 0 corresponds to the *Pnma* phase while η = 1 to the *Immm* (a) or *Cmcm* (b) phase. (c) Possible decay channels for the *Immm* phase: *Immm* → *Pnma*, *Immm* → *Cmcm*, and *Immm* → *Fm*3̅*m*. We recall that 1 mRy/u.c. corresponds to 40 K while 1 meV/u.c. corresponds to 3 K.

Considering the *n*_*e*_ = 0.0 *e*^–^/u.c. case, both the reactions toward *Immm* and *Cmcm* present a large kinetic barrier. As the PC is increased, the *Pnma* → *Immm* barrier is gradually suppressed and becomes zero for *n*_*e*_ ≃ *n*_*e*_^*c*^. Since the lowest-energy configuration corresponds to the *Immm* phase for *n*_*e*_ > *n*_*e*_^*c*^, the *Pnma* → *Immm* reaction becomes spontaneous. Conversely, the *Pnma* → *Cmcm* barrier remains finite for every value of PC.

The question arises if the structural transformation toward the *Immm* is permanent, i.e. if the *Immm* phase remains stable at longer times after carrier recombination has taken place. To correctly describe the slow structural dynamics, one must also include volume relaxation effects.

To this aim, we consider variable-volume reaction paths in the absence of photoexcitation starting from the *Immm* structure, namely, *Immm* → *Pnma*, *Immm* → *Cmcm*, and *Immm* → *Fm*3̅*m*. The initial *Immm* structure corresponds to the photoinduced transient phase, while the final structures are obtained through variable volume optimization with zero PC. Along the reactions, both the internal coordinates and the structural parameters vary. The results of our calculations are listed in Figure 4c. Both the transformations *Immm* → *Pnma* and *Immm* → *Cmcm* present large energy barriers and thus are not spontaneous. Conversely, the *Immm* → *Fm*3̅*m* reaction does not have a barrier and can occur spontaneously. Hence, once the transient *Immm* phase has been stabilized, electron–hole recombination takes place, and the system decays into the TCI *Fm*3̅*m* phase.

In addition, we stress that a large free-energy barrier, amounting to 15 mRy/u.c., exists between the *Fm*3̅*m* and the *Pnma* (see Figure S6). Hence, the topological rocksalt phase can survive thermal fluctuations corresponding to ∼600 K before decaying into the *Pnma* phase. This finding demonstrates the occurrence of a nonthermal path stabilizing the SnSe rocksalt structure and provides a nonthermal synthesis mechanism for the rocksalt TCI phase.

We stress the fundamental role played by light-induced symmetrization. The TCI phase of rocksalt-SnSe is protected by a combination of time-reversal and *C*_4_ symmetry, the latter being absent in both the *Pnma* and *Immm* phases. Crucially, we showed that laser irradiation favors the *Pnma* → *Immm* symmetrization, allowing the crystal to access a metastable region of the phase diagram, in close proximity to the *Fm*3̅*m* structure, which is successively stabilized after the laser removal, finally restoring the cubic *C*_4_ symmetry necessary to protect the topological crystalline order.^7^

In conclusion, we have shown that ultrafast pulses can permanently transform the topologically trivial *Pnma* phase of SnSe into the TCI rocksalt phase. The mechanism is nonthermal and does not require epitaxial growth on particular substrates. This is one of the rare cases when ultrafast pulses change the topological properties of the material. We identified the transition path and evaluated its critical fluence. A strong validation for the accuracy and predictivity of our theoretical framework is the excellent agreement of our quantum anharmonic calculations in the photoexcited regime with recent pump–probe X-ray free electron-laser measurements in the low fluence regime below the transition.

Finally, we point out that our findings demonstrate that light can be used to reshape the free-energy landscape, allowing access to otherwise unreachable regions of the phase diagram. This general result is relevant for the exploration of new phases in a broad class of materials, including monochalcogenides,^49^ which are highly relevant for energy applications, insulating/semiconducting 2D materials with strong spin–orbit coupling, and, in general, all semiconducting materials in the proximity of structural instability.

## Data Availability

The data underlying this study are openly available in Zenodo at https://doi.org/10.5281/zenodo.8413390.

## Supporting Information Available

Description of the computational methods, crystal structures, and comparison of the two different cDFT approaches (PDF)
