# Supplementary material for: Light-Induced Nonthermal Phase Transition to the Topological Crystalline Insulator State in SnSe
Source: J Phys Chem Lett. 2023 Oct 11;14(41):9329–34. doi: 10.1021/acs.jpclett.3c02450 (PMC10591509; doi:10.1021/acs.jpclett.3c02450)
Supplement: Supplementary file 1 — jz3c02450_si_001.pdf [file jz3c02450_si_001.pdf]

Supporting Information: Supporting Information for "Light-Induced Non-Thermal Phase Transition To The Topological Crystalline Insulator State In SnSe"

Stefano Mocatti,* Giovanni Marini,* and Matteo Calandra*

Department of Physics, University of Trento, Via Sommarive 14, 38123 Povo, Italy

E-mail: stefano.mocatti@unitn.it; giovanni.marini-2@unitn.it; m.calandrabuonaura@unitn.it

S1 Methods

The calculations are performed by first-principles methods within density functional theory (DFT). We employ Optimized Norm-Conserving Vanderbilt pseudopotentials,[S1] including also semi-core electrons. We set the kinetic energy cutoff to 80 Ry for the plane-wave expansion of the electronic wavefunctions to converge total energy and stress tensor calculations for variable-volume optimizations, as implemented in the QUANTUM ESPRESSO (QE) distribution.[S2,S3] For the structural optimizations, the convergence threshold on the interatomic forces is set to 10^{-5} Ry/a_0 while that on the stress tensor is set to 0.1 kbar. The exchange-correlation energy is approximated within the generalized gradient approximation (GGA), in the form proposed by Perdew, Burke and Ernzerhof (PBE).[S4]

To account for the presence of important interlayer forces, we include van der Waals correc-

tion within the exchange and correlation functional in the semi-empirical form as proposed by S. Grimme *et al.*[S5] We verify that the inclusion of vdW corrections accounts for the correct energetics of the system, correctly predicting ground state structure and improving the agreement between the calculated and measured structural parameters. It is worth verifying if the inclusion of vdW corrections affects only the predicted equilibrium volume or if it also changes substantially the phonon frequencies. To investigate this aspect we decided to compare the phonon spectrum of the ground state *Pnma* within GGA with the inclusion of van der Waals corrections and that calculated without van der Waals effects. The van der Waals corrections have been included through the Grimme-D3[S5] parametrization. The result of the calculation is displayed in Fig. S1(a). The changes to the phonon frequencies are mild and can be neglected in all the following analysis.

A $4 \times 12 \times 12$ Monkhorst-Pack[S6] k-vector grid is used to compute integrals over the Brillouin zone (BZ) for all the four structures considered. The choice of the present parameters guarantees an accuracy larger than 1 meV/atom for the calculation of the total energy. When spin-orbit coupling (SOC) is included in the calculation of the band structure of the *Immm* and $Fm\bar{3}m$ structures it gives rise to a gap opening. We note that SOC affects mildly the structural properties of the system, hence we neglect it for all the phonon spectrum calculations. For the computation of the band structure, we verify that the inclusion of many-body effects within GW amounts only to a rigid shift of the valence and conduction bands, increasing the fundamental gap. In Fig. S1 we display the calculated band structures of the orthorhombic *Pnma* phase of SnSe in the absence of photocarriers within GGA (blue curve) and with GW[S7] (black curve). From the figure, we see that the inclusion of many-body effects within GW amount to a rigid shift of the conduction and valence bands, thus increasing the fundamental gap. Hence, the inclusion of many-body effects in the GWA acts as a scissor operator and the electronic properties are well reproduced within DFT.

To simulate the effects of laser irradiation, we use the scheme presented in Ref. S8 for semiconductors and insulators. The excited photocarriers are modelled by removing a fraction x

of the valence electron and promoting them to the conduction states, in such a way that the photocarrier concentration (PC) is $n_e = x\ e^{-}/\text{u.c.}$, where u.c. stands for the unit cell of the solid. The ultrashort timescale (few ps) over which the experiment of Ref. S9 is performed, makes it reasonable to ignore physical processes related to the photocarriers recombination, which take place typically after hundreds of ps.[S10] This separation of timescales allows for a great simplification of the problem. Since the intraband thermalization timescale is usually short (hundreds of fs), the photocarriers behave effectively as a thermalized electron-hole plasma over a timescale shorter than that of photocarrier recombination. The presence of two different populations of carriers (the conduction electrons and the valence holes), requires the generalization of the electronic structure theory for the presence of two chemical potentials. These details are discussed in Ref. S8, where it is shown how to generalize the calculation of energy, forces, stress tensors and phonon frequencies in the presence of two Fermi distributions. The procedure to calculate the observables is implemented as a modification of the QE 6.6 package. The presence of two Fermi distributions requires the introduction of two smearing parameters σ_1 and σ_2. We use a Marzari-Vanderbilt smearing[S11] $\sigma_1 = \sigma_2 = 0.01$ Ry for structural optimization and phonon calculations. The electron momentum grid that grants the convergence depends strongly on the PC considered. For structural optimizations, we used mainly a $4 \times 12 \times 12$ Monkhorst-Pack grid, while for phonon calculations we employed a $7 \times 21 \times 21$ grid. Linear response calculations in this framework are performed within density-functional perturbation theory.[S8,S12] We highlight that the inclusion of dispersion forces in the total energy relies on the ground state values of Grimme-D3[S5] coefficients.

The anharmonic effects were investigated within the Stochastic self-consistent harmonic approximation (SSCHA).[S13] This approximation allows the estimation of quantum Helmholtz free energy. The Free energy is calculated ab-initio via a stochastic conjugate gradient minimization algorithm. The SSCHA requires the calculation of total energy and forces on supercells with randomly displaced atoms. We use ab initio forces in the presence of a thermalized electron-hole plasma.[S8] Once the minimum of the Free energy is reached, it

is possible to calculate the spectral function, the anharmonic phonon frequencies and the anharmonic contribution to the phonon linewidth.

For the *Pnma* calculation we choose a $2 \times 3 \times 3$ supercell containing 144 atoms for the $n_e = 0.0, 0.2\ e^-$/u.c. calculations. In the case of the *Immm* at $n_e = 0.6\ e^-$/u.c., given the presence of a 1D Peierls-like instability due to X vector nesting (see Sec."Structural and vibrational properties of photoexcited SnSe"), we choose a $6 \times 1 \times 1$ cell containing 48 atoms. The total energy, forces and stress tensor are calculated with a kinetic energy cutoff of 50 Ry. A $2 \times 5 \times 5$ Monkhorst-Pack grid is employed for $n_e = 0.0, 0.2\ e^-$/u.c. while a $1 \times 10 \times 10$ grid is used for $n_e = 0.6\ e^-$/u.c.. In the case of photoexcited calculations, we use a Marzari-Vanderbilt smearing $\sigma = 0.02$ Ry for both the electrons and holes. The free energy is minimized via a stochastic algorithm that uses the energies, the interatomic forces and the stress tensors of an ensemble of supercells. Each ensemble consists of 200 elements, with exceptions for the last one which contains 2000 elements. The minimization of the free energy takes into account both the SSCHA frequencies and the centroid positions. The free energy is minimized via a stochastic conjugate gradient algorithm with importance sampling until either the Kong-Liu effective sample size N_{eff} gets below a threshold or the free energy reaches a minimum. In the present calculation, we set $N_{eff} = 0.3$. The free energy is minimized at 0 K for both the ground state and photoexcited state.
For the visualization of the phonon eigenvectors we use the software XCrySDen,[S14] while structure visualization is performed with VESTA3 software.[S15]

S2 Ground state crystal structures

Each of the four crystal structures considered can be described by fixing seven parameters: $a, b, c, x_{Sn}, z_{Sn}, x_{Se}, z_{Se}$. The parameters a, b and c determine the orthorhombic conventional cell while x_{Sn}, z_{Sn} and x_{Se}, z_{Se} fix the Wyckoff coordinates of the tin and selenium atoms respectively. As for the y Wyckoff coordinates, they are fixed by symmetry constraints.

Each orthorhombic unit cell contains eight atoms, whose positions can be determined from the Wyckoff coordinates through Tab.S1. The values of the seven parameters listed before determine the crystal structure among the four considered. In Tab.S1 we display the values of the parameters that define each structure. As a general remark, the more constraints on the parameters, the more symmetry operations are associated with the structure. In particular, the rock-salt $Fm\bar{3}m$ has cubic symmetry, even though it is described in terms of a conventional orthorhombic cell.

The accuracy of the exchange and correlation kernel is tested by calculating the equilibrium parameters, the band structure and the phonon frequencies in the absence of photocarriers and at low temperatures. This is done by optimizing the structure via variable-volume optimization. Then, the band structure and the phonon spectrum of the equilibrium phase are calculated. For completeness, the structural and vibrational ground state properties are calculated for each structure.

In Tab.S2 we list our DFT predictions for the structural parameters and the phonon frequencies of the four A_g modes at Γ (see Fig. S3) for the $Pnma$ structure. Together with our estimates, we report the results of other DFT calculations within LDA[S16] and PBE[S16,S17] as well as experimental data.[S9,S18] We also calculated the phonon frequencies of the A_g modes including anharmonic corrections at $T = 0$ K within the SSCHA. The corresponding values are $\omega_{A_g} = 30.90, 59.21, 134.50, 142.40$ cm^{-1} for the $A_{g,1}, A_{g,2}, A_{g,3}$ and $A_{g,4}$ modes respectively. The description of the structural and vibrational properties within DFT is accurate and comparable with that of other results in the literature.

We also calculate the equilibrium parameters and Wyckoff positions for the $Cmcm$ structure at low temperatures. Just as a reference, our results can be compared with experimental data regarding the high-temperature phase of SnSe at 813 K, even though the comparison is not completely legitimate as no temperature effect is considered in the treatment. Within PBE, we obtain $a = 11.857$ Å, $b = 4.254$ Å, $c = 4.275$ Å and $x_{Sn} = 0.1258$ Å, $x_{Se} = 0.357$ Å. To be compared with experimental data collected at 813 K,[S18] namely $a = 11.713$ Å,

$b = c = 4.306$ Å and $x_{Sn} = 0.125$ and $x_{Se} = 0.357$. The equilibrium cell parameters were calculated also for the *Immm* and $Fm\bar{3}m$ structures. In doing so, we discover that the two phases are nearly degenerate. The total energy difference between the two is in fact of the order of 1 meV/u.c.. This result suggests that, at low temperatures, the *Immm* structure's lowest energy configuration corresponds to the rock-salt phase. This is consistent with the experimental observations: while the rock-salt phase exists as a metastable phase of SnSe,[S19] the *Immm* structure has never been observed for this compound. The *Immm* phase at low temperatures, coinciding with the rock-salt $Fm\bar{3}m$, is hence characterized by only one parameter a, that is the length of the cubic cell of the lattice. Our DFT calculations predict $a = 6.01$ Å while experiments[S19] show $a = 5.99$ Å.

In Fig.S2 we display the band structure and density of states (DOS) for the *Pnma*,*Cmcm* and $Fm\bar{3}m$ calculated with DFT at 0 K. Note that we included SOC for the calculation of the rock-salt band structure, which results in the opening of a band gap. Our results compare qualitatively well with other reference results from the literature.[S9] The calculated direct and indirect band gaps for the *Pnma* phase are $\Delta_d = 0.892$ eV and $\Delta_i = 0.517$ eV while the experimental measurements[S20] yield $\Delta_d = 1.3$ eV and $\Delta_i = 0.896$ eV. For the *Cmcm* structure, we predict a direct band gap $\Delta_d = 0.307$ eV while a GW calculation[S7] yields $\Delta_d = 0.464$ eV. As for the rock-salt structure, we calculated $\Delta_d = 0.211$ eV. For the calculations of DOS and band structure, we employed the tetrahedron method[S21] with a $6 \times 18 \times 18$ Monkhorst-Pack grid.

Finally, we calculate the phonon spectra of the three structures and the phonon density of states (pDOS) at the harmonic level. The phonon frequencies are calculated with reference to the primitive cell of each of the three structures. Hence, an orthorhombic cell was chosen for the *Pnma*, a monoclinic cell for *Cmcm* and a rhombohedral cell for the rock-salt. For the present calculation, we use a 50 Ry kinetic energy cutoff, a $3 \times 9 \times 9$ Monkhorst-Pack grid for the *Pnma*, a $20 \times 20 \times 10$ grid for the *Cmcm* and a $24 \times 24 \times 24$ grid for the rock-salt. The SOC is not included in the calculation as we verified that its inclusion changes mildly

the phonon frequencies. Due to the semimetallic nature of the rock-salt without SOC, we employed a Marzari-Vanderbilt smearing of 0.005 Ry. The result of our calculations is displayed in Fig.S3. Together with the phonon frequencies and pDOS relative to the *Pnma* structure, we also display experimental data obtained through inelastic neutron scattering (INS)[S16,S22] and optical measurements[S23] at 300 K. Overall, the agreement between theory and experiment is good. The LO-TO splitting of the optical branches has been included for the *Pnma* and *Cmcm* spectra via the calculation of effective charges. Note that while both *Pnma* and $Fm\bar{3}m$ are dynamically stable, the *Cmcm* phase is not stable at the harmonic level as its spectrum contains imaginary frequencies.

S3 Structural and vibrational properties at non-zero photocarrier concentration.

S3.1 Differences and comparison between cDFT methods

As we compare the results of our cDFT calculations with those obtained within cDFT as reported in Ref. S9, we decide to explain the main differences between the two approaches. Both methods aim to model the effect of photoexcited electrons by removing a fraction of carriers from the valence states and placing them in the conduction states.

The authors of Ref. S9 mention in the "APPENDIX C: DENSITY FUNCTIONAL THEORY" that the simulations have been performed with VASP. A recent paper[S24] in which S.W. Teitelbaum, Y. Huang, G. de la Peña, and M. Trigo are present as co-authors, provides some more technical details regarding their cDFT calculations. We report here a piece of text extracted from the Supplemental Material:

"Constraining the electron occupation of valence and conduction bands.

The approach is as follows:

1) Run two self-consistent calculations with the number of electrons set as the desired electron- and hole-doped cases and get the electron occupation at each k-point.

2) Manually set the number of electrons at each k-point for each band using the electron occupation computed in the first step, i.e., treat the valence band as the hole-doping case and the conduction band as the electron-doping case.
3) Constrain the electron occupation in the above state and perform phonon calculation. A detailed discussion of this approach is provided in a prior study by Paillard et al. (Physical Review Letters 123, 087601 (2019)). The estimated charge population using this approach is 0.07ne, as reported in the main text."

The approach presented in Refs. S9 and S24 is crucially different from that of Refs. S25 and S8, which we recently implemented in the 7.2 version of QUANTUM ESPRESSO. In particular, in Ref. S8 it is explained that the electron-hole occupations are distributed according to two Fermi-Dirac distributions. Moreover, the occupation numbers are determined in a self-consistent way during the computation of the total energy, allowing them to vary. This approach reflects the physical situation in which the intraband scattering time is small compared to both the phonon periods and the photocarrier recombination timescales.

Under these hypotheses, the carriers within the same group of bands can thermalize, thus yielding a quasi-equilibrium Fermi-Dirac distribution of the occupation numbers. On the other hand, there is no physical justification for fixing the population numbers in the first iteration from the outset as done in Ref. S9 and keeping them fixed from the first iteration. As a representative example, consider the case of a structural optimization calculation. Suppose that, during the initial run, the carriers' occupation numbers have been fixed as schematically shown in the left panel of Fig. S4(a). Hence, the occupation of conduction (valence) electrons will be maximum (minimum) at the bottom (top) of the conduction (valence) band. In general, during the optimization, the geometry of the cell changes, and thus also the band structure changes. In Fig.S4 the band deformation mimics that of the transition from the $Pnma$ to the $Immm$, see Fig.3(a,b) in the main text. However, since

the occupation numbers are fixed for every k-point of the grid, a deformation of the band structure can yield a distribution of carriers where the occupation of conduction (valence) electrons is not maximum (minimum) at the bottom (top) of the conduction (valence) band, see the right panel of Fig. S4(a).

Conversely, if the occupation of carriers is determined self-consistently through a Fermi-Dirac distribution (as done in Refs. S25, S8 and in our implementation) a deformation of the bands does not yield unphysical occupations as the new values are recalculated according to the new Fermi-Dirac distributions (see Fig. S4(b)). From this argument, we believe that the results relative to a calculation with fixed occupations as that done in Refs. S9 and S24 do not lie on solid physical grounds, hence cannot be trusted. On the contrary, it is legitimate to employ two Fermi-Dirac distributions as long as the interband thermalization timescale is very short.

S3.2 Structural distortion, energy barriers and phonons

We calculate the equilibrium configuration of photoexcited tin selenide for several values of PC up to 0.8 e^{-}/u.c.. The structural optimization calculations are performed both at fixed and variable volume. In the case of fixed volume optimizations, we set the cell parameters to the ground state equilibrium values. This is done to reproduce the experimental conditions, assuming that the volume has no time to change in the timescale associated with the optical measurements. In Fig.S5 we display the Wyckoff position of tin as a function of the PC. For the variable volume optimizations, we also report the equilibrium cell parameters. Together with our calculations, we report also the experimental measurements of Huang *et. al.*[S9] and their theoretical predictions obtained with the hole-doping (HD) method and with their simplified version of c-DFT. The experimental atomic displacements are reconstructed from the reduced mode amplitudes α_i, reported in Ref. S9, and the phonon eigendisplacements

$\mathbf{u}_i^s$ of the mode $A_{g,i}$ and atom s through the formula

$$\Delta \mathbf{r}_s = \sum_{i=1}^{4} \alpha_i \mathbf{u}_i^s.$$

In the current reconstruction, the contribution of the mode $A_{g,3}$ is neglected as α_3 is significantly smaller than the others. The authors estimated that 0.60 pairs/u.c. correspond to a fluence of 2.2 mJ/cm^2. We stick to their estimate and report faithfully their theoretical data in the graph. It is clear from the figure that both hole-doping and their version of c-DFT are unable to reproduce quantitatively the structural distortion. Conversely, our c-DFT implementation[S8] reproduces the experimental data with remarkable accuracy. Note also that, independently of the volume constraint, our calculation predicts a phase transition to the *Immm* structure for a critical PC corresponding to $n_e^c \simeq 0.6\ e^-$/u.c.. The atomic distortion is reproduced by c-DFT only for fixed volume structural optimizations, suggesting that volume relaxation takes place on timescales longer than those of the experimental measurements of Ref. S9.

To observe how the energy barriers of the reactions *Pnma*→*Immm* and *Pnma*→*Cmcm* change with PC, we evaluate the total energy along linear paths connecting the initial and final phase. The transformation has been parametrized with a parameter η in the following way

$$C_i(\eta) = C_i(\eta = 0) + \eta[C_i(\eta = 1) - C_i(\eta = 0)],\ \eta \in [0, 1],$$

where $C_i = \{x_{Sn}, z_{Sn}, x_{Se}, z_{Se}\}$ and $\eta = 0$ corresponds to the *Pnma* while $\eta = 1$ to the *Cmcm/Immm* depending on the reaction considered. We study the reaction both at fixed and variable volume. In the case of variable volume reactions, we include also a, b and c among the variables C_i. The values of the C_i for $\eta = 0, 1$ for any PC, correspond to the optimal parameters of the associated structure, obtained via fixed or variable volume optimization. The total energy is calculated for values of PC ranging from 0 up to 0.8 e^-/u.c.. In Fig.S6 we report the obtained energy profile for fixed and variable volume reactions. The

fixed volume reactions show that, while the *Pnma*→*Cmcm* transformation is forbidden for every value of PC, the *Pnma*→*Immm* reaction becomes spontaneous for $n_e \gtrsim 0.55$ e^-/u.c.. Note also that the second derivative of energy profile about the *Cmcm* phase is negative for all values of n_e. This suggests that the *Cmcm* phase presents imaginary phonons for all values of n_e and thus cannot be stabilized with light. So, the solid spontaneously relaxes to the *Immm* phase for PC greater than the critical value.

Similar conclusions can be drawn by looking at the plot corresponding to variable volume transformations. The only difference is that *Pnma*→*Cmcm* is not forbidden even though the reaction *Pnma*→*Immm* is energetically more favourable. We also stress that the energy barrier of the variable volume reaction $Pnma \rightarrow Immmm$ becomes zero for $n_e \sim n_e^c$ even though the $Immm$ phase becomes energetically favoured for smaller values of the PC. As a further remark, we recall that at zero PC the $Immm$ spontaneously recovers all the $Fm\bar{3}m$ symmetries, see Tab.S3. Hence, the variable volume reaction $Pnma \rightarrow Immm$ at zero PC represents actually the $Pnma \rightarrow Fm\bar{3}m$ reaction, confirming the meta-stability of the rock-salt structure in absence of photocarriers.

The form of the energy profile suggests that the phase transition connecting the *Pnma* and *Immm* phases is of the first order kind. This can be understood by recalling that the energy coincides with the free energy at zero temperature. Moreover, if η is considered as the order parameter, it is easy to see that its value corresponding to the minimum free energy changes discontinuously when $n_e \sim n_e^c$. The energy profile further highlights that ultrafast radiation can be used to stabilise the *Immm* structure at low temperatures. A pictorial scheme that shows how to stabilize the *Immm* phase is displayed in Fig.S6. It is sufficient to irradiate with a high fluence ultrafast laser source the sample to favour the *Pnma*→*Immm* reaction. Then, since the *Immm* phase is a local minimum of the energy for every value of PC, if the light source is removed and the sample is kept at zero temperature, the solid will end up in a metastable *Immm* structure. At zero temperature, the *Immm* phase admits a lower energy configuration corresponding to the rock-salt structure, where the lattice parameters

are constrained to be $a = 2\sqrt{2}b$ and $b = c$. Hence, through a variable volume relaxation, the solid transforms into a rock-salt, thus yielding a topological insulating $Fm\bar{3}m$ phase. This is verified through variable volume optimizations, employing PBE and LDA with the addition of van der Waals (vdW) interactions. As a reference, we report the energy difference between the ground state $Immm$ and rock-salt structures ΔE and the lattice parameters of the $Immm$ phase obtained through variable-volume optimizations in Tab.S3. The table shows that the two structures are indeed the same at zero temperatures i.e. the $Immm$ phase cannot be observed at zero temperature.

We calculate the photoexcited vibrational frequencies both at the Γ point and over the Brillouin zone. We remark that the good agreement between our calculated phonon frequencies and the experimental ones establishes a mapping among the experimental fluences and the photocarrier concentration that will be useful in future experiments. For example, the A_g frequency relative to $n_e = 0.1\ e^-$/u.c. corresponds to an experimental fluence of 0.8095 mJ/cm^2.

We can check the consistency of the present estimate through a simple calculation based on the energy absorbed by the sample. We assume that the flux $\Phi(x, y, z, t)$ of incoming energy is a function of time t, the coordinates parallel to the surface x and y and the coordinate normal to the surface z. Hence, the total absorbed energy can be written in the following form

$$E = \int dz \int dt \int dxdy \Phi(x, y, z, t) \times (1 - R),$$

where the factor (1-R) has been inserted to account for the portion of reflected light. We neglect the fraction of transmitted light since the experiment is performed on macroscopic single crystals and almost no light is transmitted through the sample.[S9] In terms of fluence, the time dependence of the pulse has already been integrated out, hence we can ignore the integral in time. Moreover, we can remove the integrals in x, y if we substitute the flux with

the fluence and multiply by the illuminated area A. We are left with the following equation

$$E = \int dz f(z) \times (1 - R)A.$$

We can assume that the fluence has a simple exponential dependence in z: $f(z) = f_0 \exp(-\alpha z)$, where α is the absorption coefficient. The fraction of energy absorbed by the sample at a penetration depth equal to α^{-1} is

$$\frac{\int_0^{\alpha^{-1}} dz f_0 e^{-\alpha z}}{\int_0^{+\infty} dz f_0 e^{-\alpha z}} = (1 - e^{-1}) = \frac{e-1}{e}.$$

The absorbed energy per unit of area and penetration length takes thus the form

$$\frac{E}{Az} = \frac{f_0(1-R)}{\alpha^{-1}} \frac{e-1}{e}.$$

By multiplying both sides of the equation by the unit cell volume Ω_0, we obtain the expression for the absorbed energy per unit cell

$$E/u.c. = \frac{f_0(1-R)\Omega_0}{\alpha^{-1}} \frac{e-1}{e}.$$

We recall that the absorbed energy is equal to the number of absorbed photons times the quantum of energy $\hbar\omega$. We assume that for each absorbed photon, an electron is excited in the conduction bands. This assumption is reasonable as long as the quantum of energy is larger than the fundamental gap and the photocarrier concentration is not too large. With this assumption, we finally obtain an expression for the photocarrier concentration $n_e = N/u.c.$

$$n_e = \frac{f_0(1-R(\omega))\Omega_0}{\alpha^{-1}(\omega)\hbar\omega} \frac{e-1}{e},$$

where we have explicitly written the dependence on ω of the reflective index R and the absorption coefficient α. The present expression depends only on the optical properties of

the sample and is thus independent of the specific experimental setup. We can evaluate it to find the conversion factor between f_0 and n_e and see if it matches the one determined before. The experimental value of the absorption coefficient is $\alpha = 1.75 \times 10^5$ cm^{-1} while that of the reflectivity is $R \lesssim 0.1$.[S26] The calculated unit cell volume is $\Omega_0 = 216$ Å^3 while the photon energy is $\hbar\omega = 1.55$ eV.[S9] By plugging these values in the previous equation we obtain that 1 mJ/cm^2 corresponds to 0.085 e^-/u.c., while by matching the harmonic phonon frequencies we obtain that 1 mJ/cm^2 corresponds to 0.123 e$^-$/u.c.. The two estimates are comparable, hence we validate the fluence to PC conversion through phonon frequency matching.

The full harmonic phonon spectrum is calculated for a few different values of PC. In Fig.S7(a) we report the phonon spectrum along a path and the phonon DOS (pDOS). As it can be observed, the presence of an electron-hole plasma results in the softening of all the frequencies. We report also the harmonic and anharmonic phonon spectrum at $T = 0$ K for a low value of the PC, corresponding to $n_e = 0.2$ e^-/u.c., see Fig. S7(b). The inset shows the comparison between the calculated phonon frequencies and recent experimental data obtained from time-resolved diffusive X-ray scattering.[S27]

For values of PC greater than n_e^c, the phonon frequency of one mode becomes imaginary, in correspondence to the X point. The presence of imaginary frequencies signals the presence of dynamical instability. The structure corresponding to a lower value of the energy can be found by moving the atoms according to the eigenvector of the unstable mode. To do this, we consider a $2 \times 1 \times 1$ supercell where the modes at X fold back to the Γ point. The phonon eigenvector corresponding to the imaginary mode at Γ is represented pictorially in Fig. S7(d). This mode shifts primarily the selenium atoms along z with a $2 \times 1 \times 1$ periodicity. The atoms are then shifted according to the form of the eigenvector and the internal coordinates are finally optimized at a fixed volume. The final structure is depicted in Fig. S7(d). The total energy gain corresponds to 1.5 meV/atom. The present structural distortion is an example of a Peierls distortion, in which the $1 \times 1 \times 1$ periodicity of the lattice is broken and the new periodic cell becomes the $2 \times 1 \times 1$ supercell. The presence of a Peierls-like instability due

to Fermi surface nesting is also evident looking at the nesting function, *i.e.* the imaginary part of the non-interacting electronic susceptibility

$$\chi_0(\mathbf{q},\omega) = \sum_{\mathbf{k},m,n} \frac{f(\epsilon_{\mathbf{k},n}) - f(\epsilon_{\mathbf{k+q},m})}{\epsilon_{\mathbf{k},n} - \epsilon_{\mathbf{k+q},m} - \omega - i\eta},$$

where η is a vanishing quantity. The presence of a peak in the nesting function at a given $\mathbf{q}$ signals a nesting of the Fermi surfaces through the $\mathbf{q}$ vector. In Fig. S7(c) we display the nesting function calculated on the k_z=0 plane of the orthorhombic Brillouin zone at zero temperature and $n_e = 0.6\ e^-$/u.c.. A proper regularization of the Dirac delta has been used. Note the presence of a peak in the proximity of the X point $\mathbf{q} = (0.5, 0, 0)$.

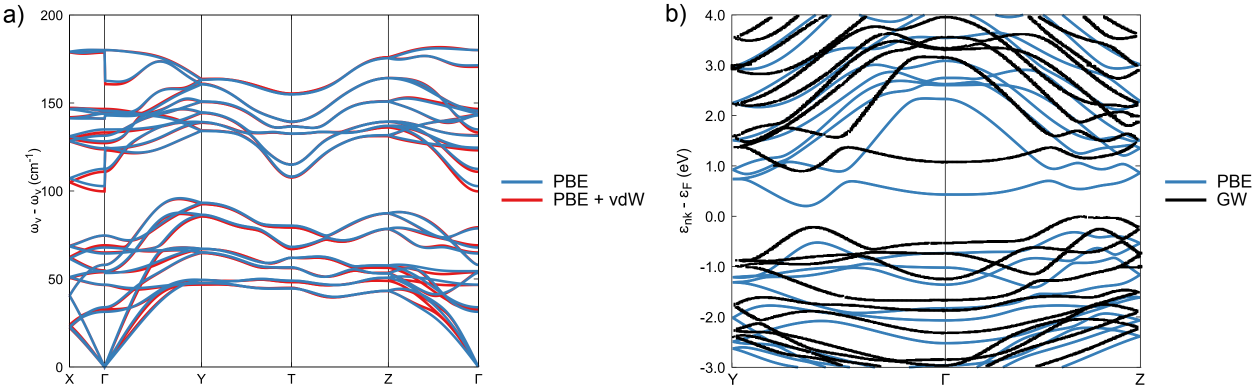

Figure S1: Panel a: Ground state phonon spectrum of the *Pnma* phase calculated with PBE (blue curves) and PBE plus van der Waals corrections (red curves). Panel b: Kohn-Sham (blue) and GW (black) band structure of *Pnma* SnSe in absence of photocarriers.

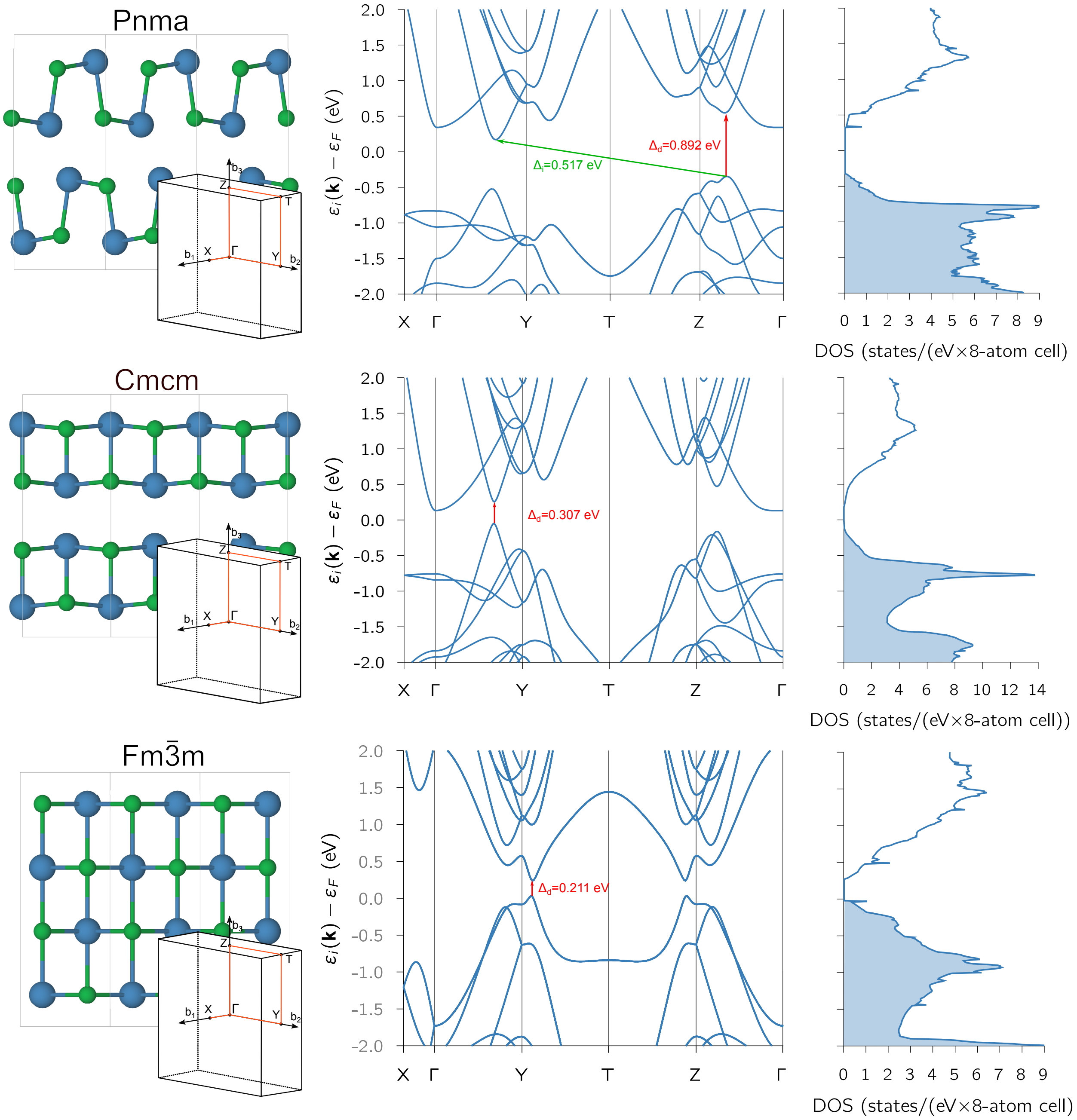

Figure S2: Ground state bandstructure and DOS for the three structures considered. The crystal structures, BZs, direct and indirect band gaps are also highlighted.

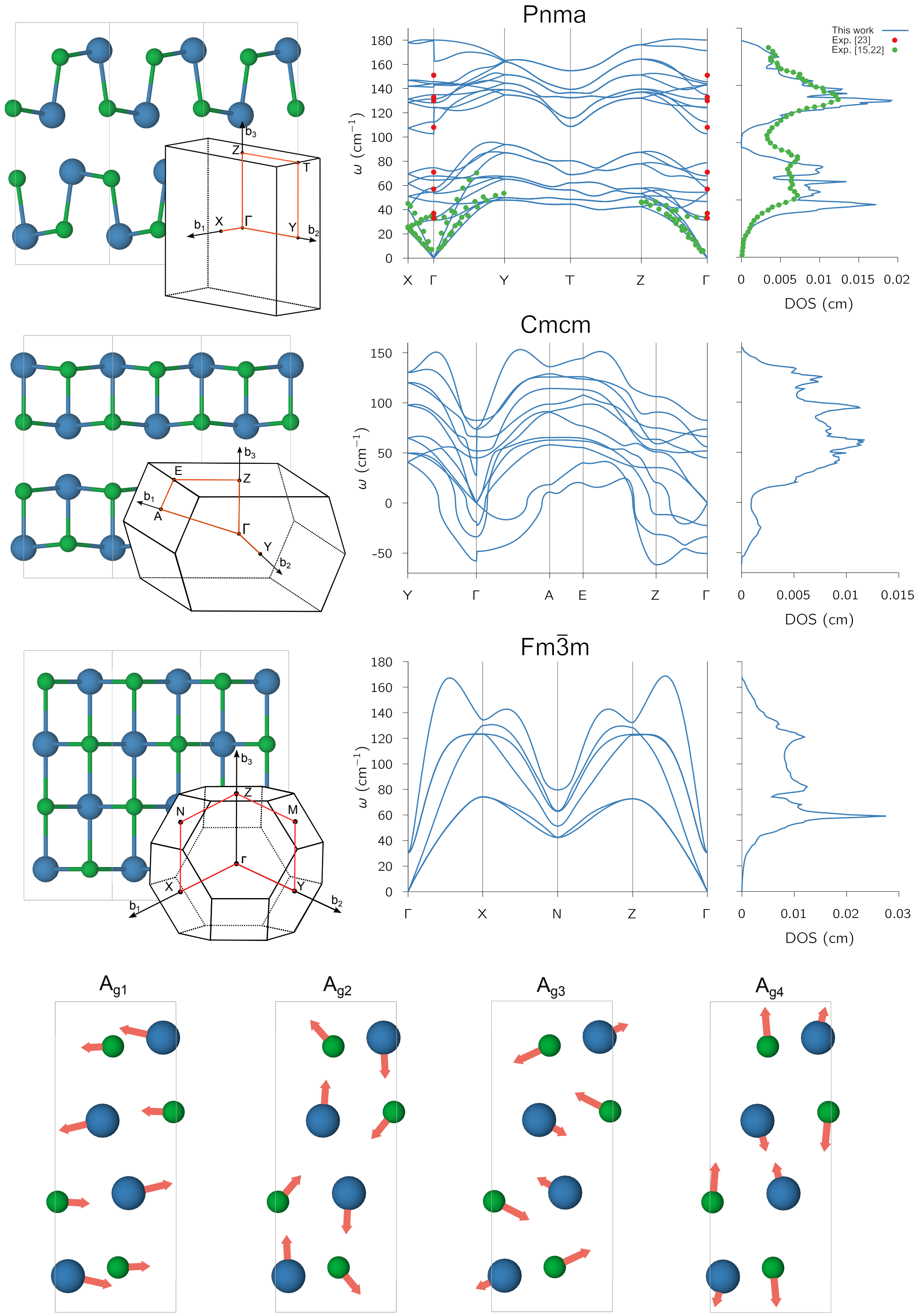

Figure S3: Ground state phonon spectra and pDOS for the three structures considered. The corresponding BZs relative to the primitive cells and the A_g modes of the *Pnma* structure are also displayed.

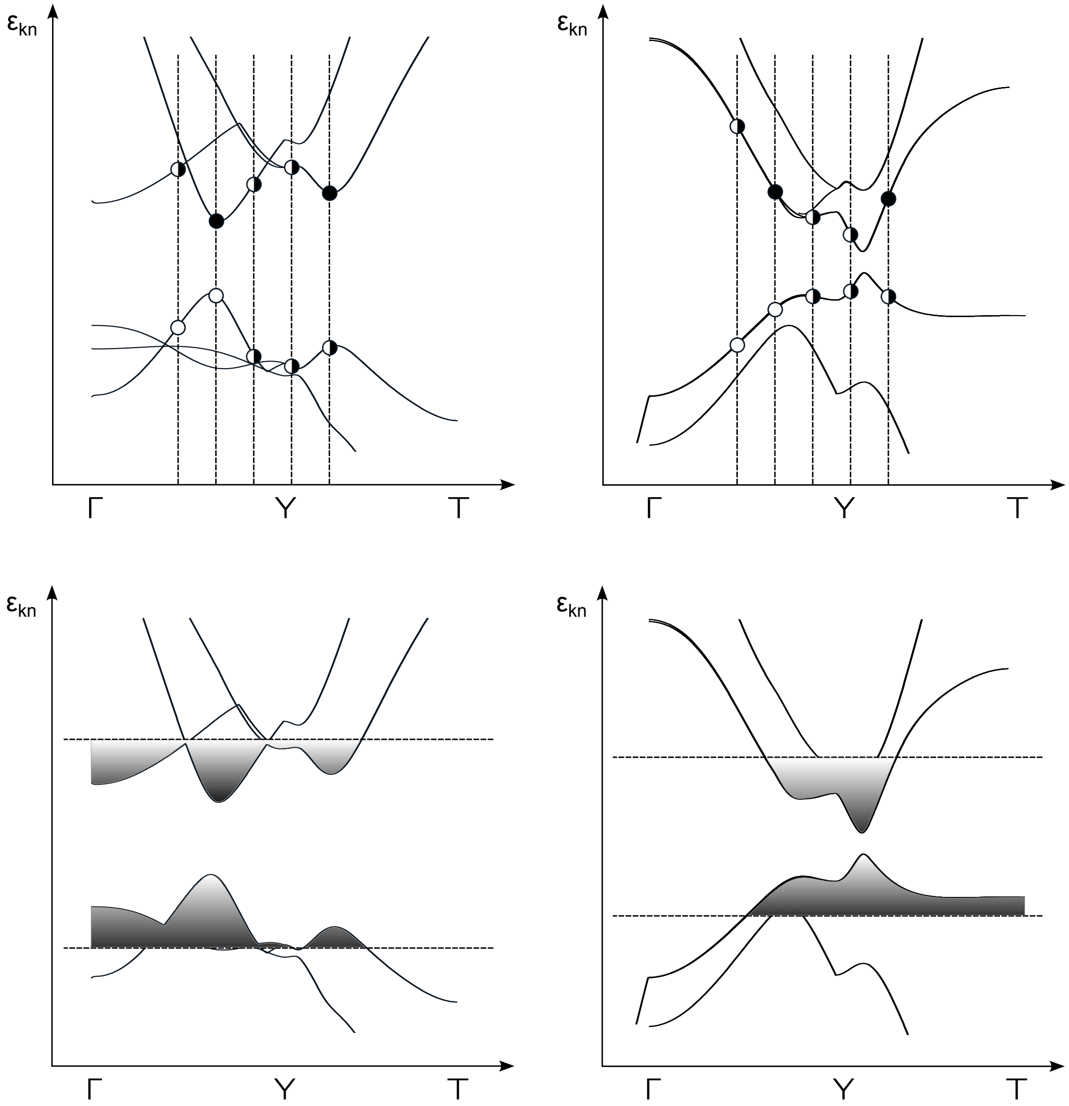

Figure S4: Comparison between the procedure carried out in Refs. S9 and S24 and the cDFT framework of Refs. S25 and S8. Top panels: the carriers' occupation numbers are calculated during an initial run and afterwards fixed (as in Refs. S9 and S24). The half-filled circle represents a non-integer number for the occupation. As it can be seen the final state of the self-consistent run result in an incorrect occupation of conduction and valence states. Panel b: the carriers' occupation numbers are calculated through a double Fermi-Dirac distribution in a self-consistent way (as in Refs. S25 and S8). Even if a band deformation occurs during the self-consistent run it is taken into account so that the right Fermi-Dirac occupation is always recovered.

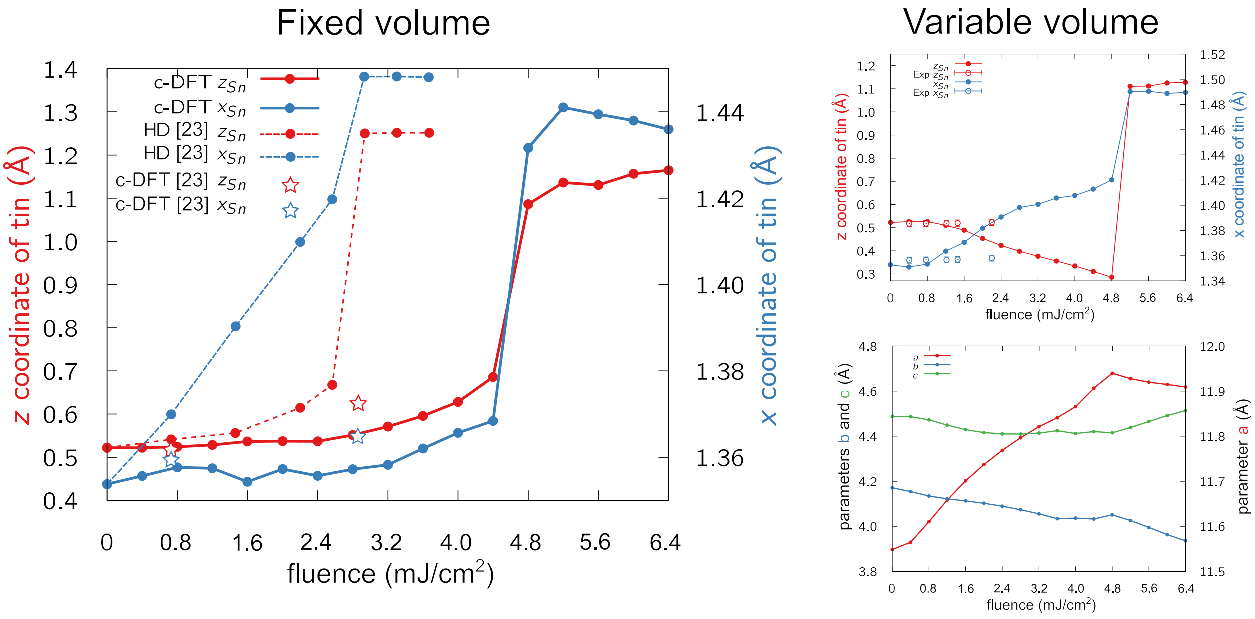

Figure S5: Wyckoff position of the tin atom as a function of the photocarrier concentration. Both the data relative to fixed and variable volume optimizations have been reported. The dashed curve in the fixed volume optimization graphs is the result of theoretical calculations with the hole-doping method as reported in Ref. S9 while the star-shaped points refer to c-DFT calculations from Ref. S9. For the variable volume calculations, also the behavior of the lattice parameters has been reported.

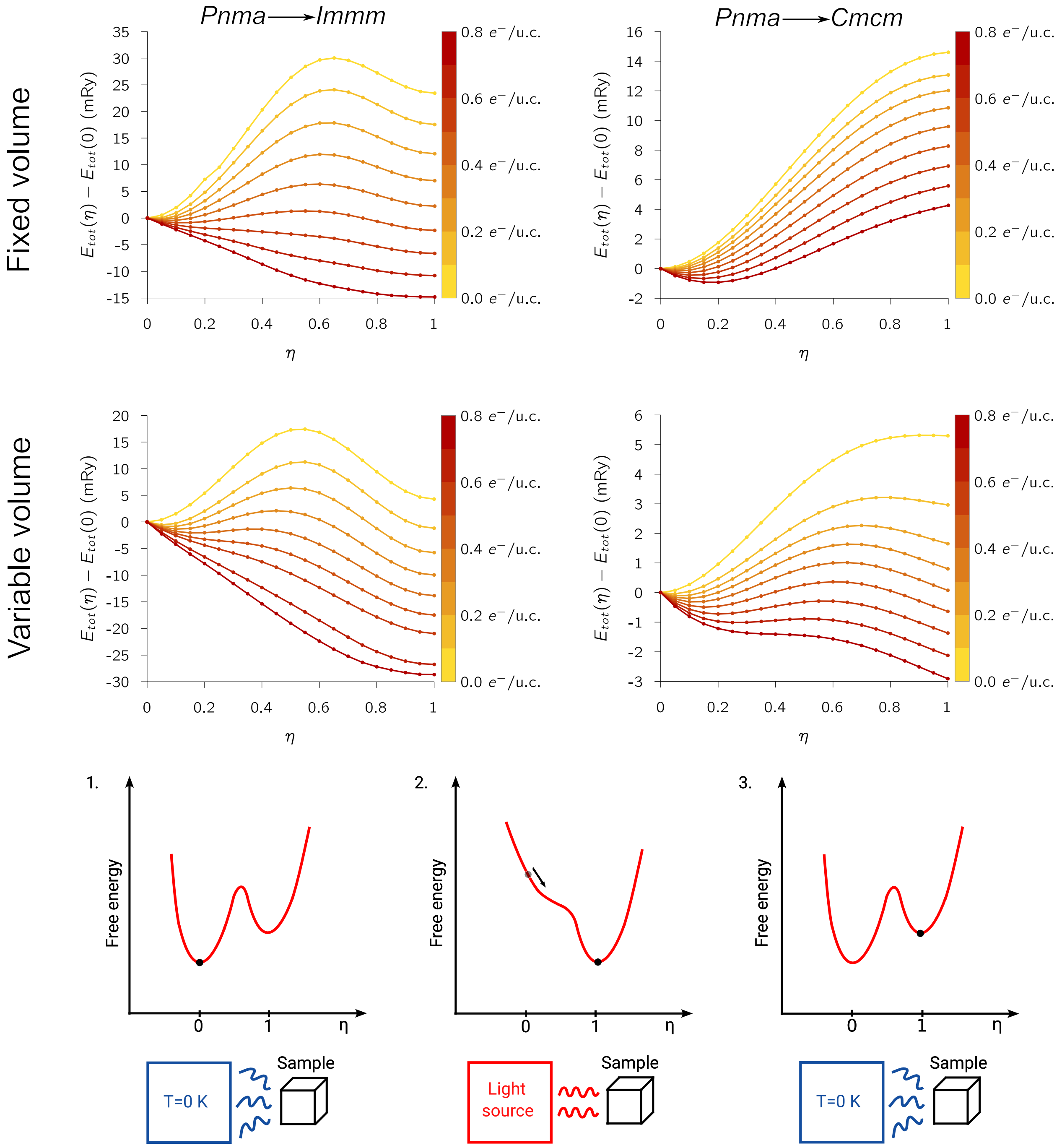

Figure S6: Fixed and variable volume energies (per unit cell) curves along a linear reaction path for $Pnma \rightarrow Immm$ and $Pnma \rightarrow Cmcm$. A scheme for the stabilization of the $Fm\bar{3}m$ structure at low temperatures with ultrafast laser irradiation is also displayed.

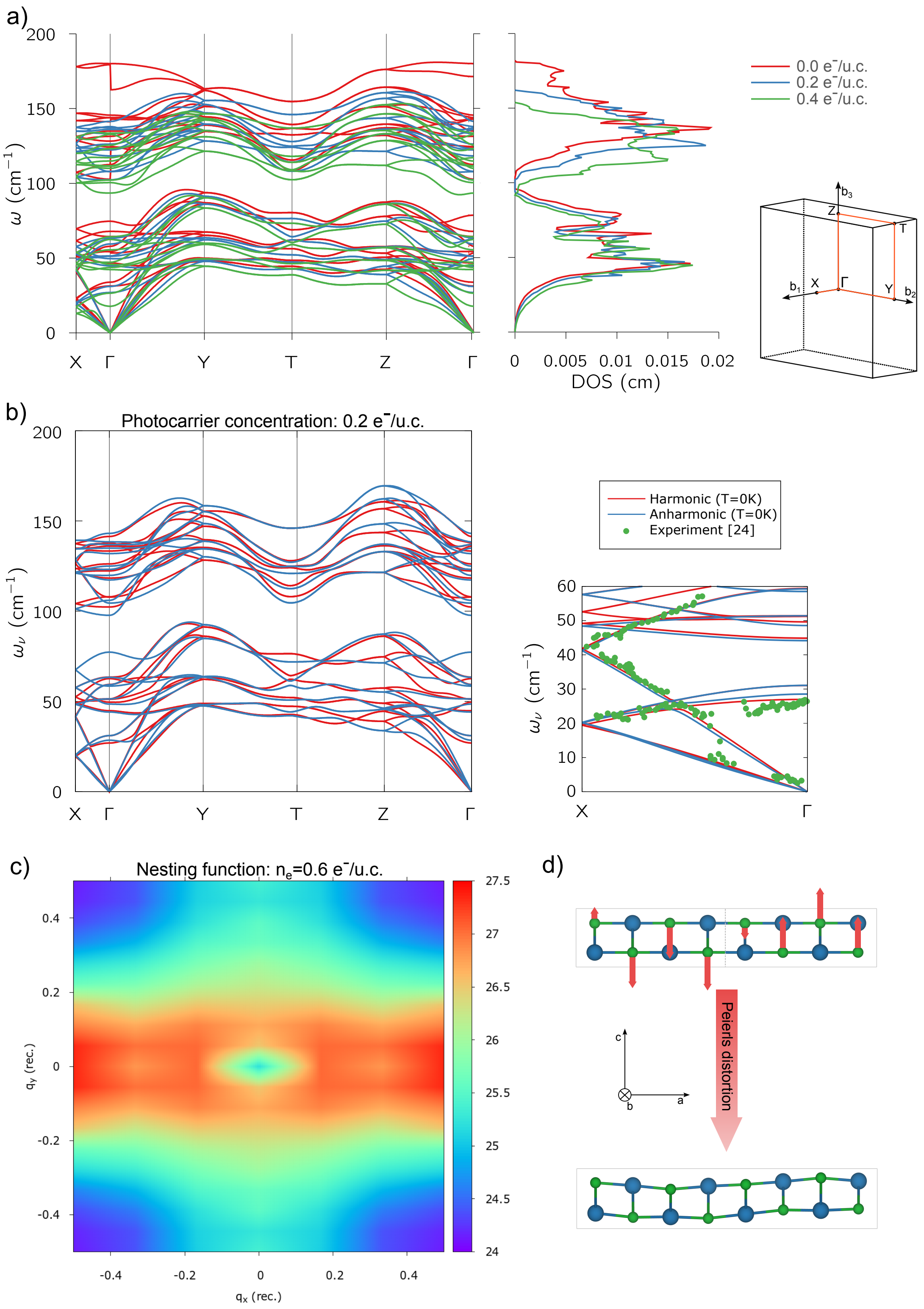

Figure S7: Panel a: full phonon spectrum and pDOS for three different values of PC below n_e^c. Panel b: harmonic and anharmonic $T = 0$ K phonon spectrum for $n_e = 0.2\ e^-$/u.c..Panel c: nesting function calculated on the XY plane of the BZ for $n_e = 0.6\ e^-$/u.c.. Panel d: schematic picture of the chain-like Peierls distortion induced by the electron-hole plasma.

Table S1: Top: Internal fractional coordinates i.e. Wyckoff positions, of the atom X (X =Sn,Se) for the $Pnma$,$Cmcm$,$Immm$ and $Fm\bar{3}m$ structures inside the conventional orthorhombic unit cell. Bottom: The table shows how the four crystal structures under examination can be described by varying at most seven parameters. The more symmetric the phase, the more constrained the parameters.

Atom	x (cryst. coord.)	y (cryst. coord.)	z (cryst. coord.)
$X_{1,2}$	x_X, $x_X + 0.5$	0.25	z_X, $0.5 - z_X$
$X_{3,4}$	$1 - x_X, 0.5 - x_X$	0.75	$0.5 + z_X, 1 - z_X$

Structure	a	b	c	x_X (cryst. coord.)	z_X (cryst. coord.)
$Pnma$	free	free	free	free	free
$Cmcm$	free	free	free	free	0.500
$Immm$	free	free	free	0.125,0.875	0.250
$Fm\bar{3}m$	free	$a/2\sqrt{2}$	$a/2\sqrt{2}$	0.125,0.875	0.250

Table S2: List of calculated and measured parameters characterizing the *Pnma* structure. The list includes the cell parameters, the Wyckoff coordinates and the phonon frequencies of the A_g modes at Γ.

Observable	Exp.[S9,S18]	LDA[S16]	LDA[S9]	PBE[S16,S17]	This work (LDA)	This work (PBE)
a (Å)	11.502	11.309	11.310	11.756	11.313	11.545
b (Å)	4.153	4.119	4.120	4.205	4.118	4.171
c (Å)	4.450	4.300	4.300	4.547	4.311	4.488
x_{Sn} (cryst. coord)	0.121	0.117	0.120	0.1206	0.117	0.117
z_{Sn} (cryst. coord)	0.106	0.091	0.090	0.1144	0.095	0.107
x_{Se} (cryst. coord)	0.355	0.358	0.090	0.355	0.357	0.355
z_{Se} (cryst. coord)	0.024	0.0247	0.0200	0.0246	0.026	0.021
$\omega_{A_{g,1}}$ (cm^{-1})	33.62			31.60	31.02	30.91
$\omega_{A_{g,2}}$ (cm^{-1})	69.10			61.50	66.57	59.77
$\omega_{A_{g,3}}$ (cm^{-1})	130.55			136.10	127.60	139.50
$\omega_{A_{g,4}}$ (cm^{-1})	150.19			160.70	154.80	142.10

Table S3: Results of the structural optimization for the *Immm* and rock-salt phases. The values in the table show that the two structures coincide at zero temperature for every choice of the DFT functional.

Exchange and correlation functional	PBE+vdW	LDA+vdW	PBE
$\|\Delta E\|/u.c.$ (mRy)	< 0.1	< 0.1	< 0.1
$\|a/b - 2\sqrt{2}\|$	$< 10^{-4}$	$< 10^{-4}$	$< 10^{-5}$
$\|b/c - 1\|$	$< 10^{-4}$	$< 10^{-5}$	$< 10^{-4}$

References

(S1) Hamann, D. R. Optimized norm-conserving Vanderbilt pseudopotentials. *Phys. Rev. B* **2013**, *88*, 085117.

(S2) Giannozzi, P.; Baroni, S.; Bonini, N.; Calandra, M.; Car, R.; Cavazzoni, C.; Ceresoli, D.; Chiarotti, G. L.; Cococcioni, M.; Dabo, I.; Corso, A. D.; de Gironcoli, S.; Fabris, S.; Fratesi, G.; Gebauer, R.; Gerstmann, U.; Gougoussis, C.; Kokalj, A.; Lazzeri, M.; Martin-Samos, L.; Marzari, N.; Mauri, F.; Mazzarello, R.; Paolini, S.; Pasquarello, A.; Paulatto, L.; Sbraccia, C.; Scandolo, S.; Sclauzero, G.; Seitsonen, A. P.; Smogunov, A.; Umari, P.; Wentzcovitch, R. M. QUANTUM ESPRESSO: a modular and open-source software project for quantum simulations of materials. *Journal of Physics: Condensed Matter* **2009**, *21*, 395502.

(S3) Giannozzi, P.; Andreussi, O.; Brumme, T.; Bunau, O.; Nardelli, M. B.; Calandra, M.; Car, R.; Cavazzoni, C.; Ceresoli, D.; Cococcioni, M.; Colonna, N.; Carnimeo, I.; Corso, A. D.; de Gironcoli, S.; Delugas, P.; DiStasio, R. A.; Ferretti, A.; Floris, A.; Fratesi, G.; Fugallo, G.; Gebauer, R.; Gerstmann, U.; Giustino, F.; Gorni, T.; Jia, J.; Kawamura, M.; Ko, H.-Y.; Kokalj, A.; Küçükbenli, E.; Lazzeri, M.; Marsili, M.; Marzari, N.; Mauri, F.; Nguyen, N. L.; Nguyen, H.-V.; de-la Roza, A. O.; Paulatto, L.; Poncé, S.; Rocca, D.; Sabatini, R.; Santra, B.; Schlipf, M.; Seitsonen, A. P.; Smogunov, A.; Timrov, I.; Thonhauser, T.; Umari, P.; Vast, N.; Wu, X.; Baroni, S.

Advanced capabilities for materials modelling with Quantum ESPRESSO. *Journal of Physics: Condensed Matter* **2017**, *29*, 465901.

(S4) Perdew, J. P.; Burke, K.; Ernzerhof, M. Generalized Gradient Approximation Made Simple. *Phys. Rev. Lett.* **1996**, *77*, 3865–3868.

(S5) Grimme, S.; Antony, J.; Ehrlich, S.; Krieg, H. A consistent and accurate ab initio parametrization of density functional dispersion correction (DFT-D) for the 94 elements H-Pu. *The Journal of Chemical Physics* **2010**, *132*, 154104.

(S6) Monkhorst, H. J.; Pack, J. D. Special points for Brillouin-zone integrations. *Phys. Rev. B* **1976**, *13*, 5188–5192.

(S7) Shi, G.; Kioupakis, E. Quasiparticle band structures and thermoelectric transport properties of p-type SnSe. *Journal of Applied Physics* **2015**, *117*, 065103.

(S8) Marini, G.; Calandra, M. Lattice dynamics of photoexcited insulators from constrained density-functional perturbation theory. *Phys. Rev. B* **2021**, *104*, 144103.

(S9) Huang, Y.; Yang, S.; Teitelbaum, S.; De la Peña, G.; Sato, T.; Chollet, M.; Zhu, D.; Niedziela, J. L.; Bansal, D.; May, A. F.; Lindenberg, A. M.; Delaire, O.; Reis, D. A.; Trigo, M. Observation of a Novel Lattice Instability in Ultrafast Photoexcited SnSe. *Phys. Rev. X* **2022**, *12*, 011029.

(S10) Sundaram, S. K.; Mazur, E. Inducing and probing non-thermal transitions in semiconductors using femtosecond laser pulses. *Nature Materials* **2002**, *1*, 217–224.

(S11) Marzari, N.; Vanderbilt, D.; De Vita, A.; Payne, M. C. Thermal Contraction and Disordering of the Al(110) Surface. *Phys. Rev. Lett.* **1999**, *82*, 3296–3299.

(S12) Baroni, S.; de Gironcoli, S.; Dal Corso, A.; Giannozzi, P. Phonons and related crystal properties from density-functional perturbation theory. *Rev. Mod. Phys.* **2001**, *73*, 515–562.

(S13) Monacelli, L.; Bianco, R.; Cherubini, M.; Calandra, M.; Errea, I.; Mauri, F. The stochastic self-consistent harmonic approximation: calculating vibrational properties of materials with full quantum and anharmonic effects. *Journal of Physics: Condensed Matter* **2021**, *33*, 363001.

(S14) Kokalj, A. Computer graphics and graphical user interfaces as tools in simulations of matter at the atomic scale. *Computational Materials Science* **2003**, *28*, 155–168, Proceedings of the Symposium on Software Development for Process and Materials Design.

(S15) Momma, K.; Izumi, F. *VESTA3* for three-dimensional visualization of crystal, volumetric and morphology data. *Journal of Applied Crystallography* **2011**, *44*, 1272–1276.

(S16) Bansal, D.; Hong, J.; Li, C. W.; May, A. F.; Porter, W.; Hu, M. Y.; Abernathy, D. L.; Delaire, O. Phonon anharmonicity and negative thermal expansion in SnSe. *Phys. Rev. B* **2016**, *94*, 054307.

(S17) Gong, X.; Wu, H.; Yang, D.; Zhang, B.; Peng, K.; Zou, H.; Guo, L.; Lu, X.; Chai, Y.; Wang, G.; Zhou, X. Temperature dependence of Raman scattering in single crystal SnSe. *Vibrational Spectroscopy* **2020**, *107*, 103034.

(S18) Adouby, K.; Perez-Vicente, C.; Jumas, J. C. Structure and temperature transformation of SnSe. Stabilization of a new cubic phase Sn4Bi2Se7. *Zeitschrift für Kristallographie - Crystalline Materials* **1998**, *213*, 343–349.

(S19) Mariano, A. N.; Chopra, K. L. POLYMORPHISM IN SOME IV-VI COMPOUNDS INDUCED BY HIGH PRESSURE AND THIN-FILM EPITAXIAL GROWTH. *Applied Physics Letters* **1967**, *10*, 282–284.

(S20) Zhao, L.-D.; Lo, S.-H.; Zhang, Y.; Sun, H.; Tan, G.; Uher, C.; Wolverton, C.; Dravid, V. P.; Kanatzidis, M. G. Ultralow thermal conductivity and high thermoelectric figure of merit in snse crystals. *Nature* **2014**, *508*, 373–377.

(S21) Blöchl, P. E.; Jepsen, O.; Andersen, O. K. Improved tetrahedron method for Brillouin-zone integrations. *Phys. Rev. B* **1994**, *49*, 16223–16233.

(S22) Li, C. W.; Hong, J.; May, A. F.; Bansal, D.; Chi, S.; Hong, T.; Ehlers, G.; Delaire, O. Orbitally driven giant phonon anharmonicity in SnSe. *Nature Physics* **2015**, *11*, 1063–1069.

(S23) Nikolić, P. M.; Milković, L.; Mihajlović, P.; Lavrenčić, B. Raman scattering in snse. *Czechoslovak Journal of Physics* **1978**, *28*, 456–459.

(S24) Krapivin, V.; Gu, M.; Hickox-Young, D.; Teitelbaum, S. W.; Huang, Y.; de la Peña, G.; Zhu, D.; Sirica, N.; Lee, M.-C.; Prasankumar, R. P.; Maznev, A. A.; Nelson, K. A.; Chollet, M.; Rondinelli, J. M.; Reis, D. A.; Trigo, M. Ultrafast Suppression of the Ferroelectric Instability in $KTaO_3$. *Phys. Rev. Lett.* **2022**, *129*, 127601.

(S25) Tangney, P.; Fahy, S. Density-functional theory approach to ultrafast laser excitation of semiconductors: Application to the A_1 phonon in tellurium. *Phys. Rev. B* **2002**, *65*, 054302.

(S26) Guo, Z.; Gu, H.; Yu, Y.; Wei, Z.; Liu, S. Broadband and Incident-Angle-Modulation Near-Infrared Polarizers Based on Optically Anisotropic SnSe. *Nanomaterials* **2023**, *13*.

(S27) Huang, Y.; Teitelbaum, S.; Yang, S.; na, G. D. l. P.; Chollet, T. S. M.; Zhu, D.; Niedziela, J. L.; Bansal, D.; May, A. F.; Lindenberg, A. M.; Delaire, O.; Trigo, M.; Reis, D. A. Determination of nonthermal bonding origin of a novel photoexcited lattice instability in SnSe. 2023; `https://arxiv.org/abs/2301.08955`.
